# A New Era for Poor-Law Nurses

**Published:** 1920-12-11

**Authors:** 


### THE MATRONS' AND SISTERS' DEPARTMENT.

### A NEW ERA FOR POOR-LAW NURSES.

The proposal of Dr. Addison to sanction the use of Poor-law infirmaries for the reception of paying patients is likely to have a far-reaching effect on the nursing service of these institutions. Apart from such fine establishments as London and other great cities can boast among their municipal hospitals, Poor-law infirmaries often labour under two unfortunate impediments to success. They are managed for the most part by persons who do not understand the first principles of efficiency in institutions for the sick, and they are still resorted to under something like compulsion by patients who believe themselves to be losing caste by accepting their advantages. Our columns bear frequent witness to the first-named impediment, for it is usually the nursing staff which suffers when guardians mismanage sick wards or infirmary. But the attitude of the patients is even more deplorable. While it continues, and in many places reluctance to enter the infirmary is very strongly marked, one of the main purposes of these costly institutions is defeated; some who most need medical and nursing care will linger out their days deprived of the alleviations which the infirmary exists to supply.

Under a system of paying patients there is every hope that drastic changes will be made in the direction both of workhouse sick wards and of infirmaries under the charge of a residential medical officer. Wherever the Poor-law infirmary has approximated to the status of a voluntary hospital this has happened through the adoption of the system which has brought the voluntary hospitals of the country into such good repute. There is found a training-school for nurses under the sway of a matron of wide experience, able to command the confidence of resident medical officer and of her whole staff. She receives the ungrudging support of the guardians who appointed her and the nursing committee which controls the whole establishment. This is the corner-stone on which efficient nursing must rest, and without efficient nursing the whole establishment must go to pieces.

It is for lack of ability and liberality to secure the right woman as matron, no less than lack of generous support and backing when they have found her, that Poor-law "scandals" arise, that nurses are hard to obtain, discipline is slack, changes frequent, and a general air of unrest observable. Even when the matron is efficient her hands are often tied by those in authority to an extent which few can appreciate who have not themselves taken office under the Poor-law.

Before the war, when it was possible to calculate to within threepence a head a week what it should cost to supply board to resident workers, the cost of food in Poor-law establishments averaged a third more than in voluntary hospitals, and the result was generally far less satisfactory. Monotony was inevitable to the system in use, the cooking was inferior, the quality was far below what could be procured for the same money in open market, and no one was satisfied. This was the result of a system which allowed the matron no control over the commissariat and no power to add even a lettuce to nurses' supper without a formal requisition to the board. With the transformation of Poor-law infirmaries into State hospitals, the first reform to be undertaken should be that of the messing arrangements.

We foresee a great improvement in status for the nurses trained in the State hospitals. With an eight-hour day and the improvement in general conditions which usually goes hand in hand with more leave, it cannot be long before the position of probationer in these institutions becomes eagerly sought for by the right kind of young woman. We look for a more rational and just pension scheme to retain them in the service. We look for the growth of legitimate pride in their training-schools, and high associations connected with their Alma Mater such as are observable in the Poor-law infirmaries which already lead the way. And we look for one feature in addition.

Public-health nursing is the nursing of the future. Where should it expand its roots better than in public infirmaries designed to raise the standard of health in the community? It ought to be a feature of these establishments, a distinguishing mark of their nurse training-schools, to prepare their students for the duties of public-health nurses.

We earnestly trust that the claim of the poor to contribute for their maintenance will not be overlooked. These new paying patients might well bring a new spirit into the establishment. If all pay, as now in voluntary hospitals, according to means, the last remnant of the pauper spirit will be exorcised.

